# Functional connectivity changes in the delta frequency band following trauma treatment in complex trauma and dissociative disorder patients

**DOI:** 10.3389/fpsyt.2022.889560

**Published:** 2022-07-25

**Authors:** Yolanda R. Schlumpf, Ellert R. S. Nijenhuis, Carina Klein, Lutz Jäncke, Silke Bachmann

**Affiliations:** ^1^Division of Neuropsychology, Department of Psychology, University of Zurich, Zurich, Switzerland; ^2^Clienia Littenheid AG, Hospital for Psychiatry and Psychotherapy, Littenheid, Switzerland; ^3^Research Unit for Plasticity and Learning of the Healthy Aging Brain, University of Zurich, Zurich, Switzerland; ^4^Department of Psychiatry, Psychotherapy, and Psychosomatics, University Hospitals and University of Halle (Saale), Halle, Germany; ^5^Department of Psychiatry, University Hospitals of Geneva, Geneva, Switzerland

**Keywords:** complex trauma, dissociation, trauma treatment, electroencephalography, functional connectivity

## Abstract

**Objective:**

Phase-oriented trauma treatment is efficacious in the treatment of complex trauma and dissociative disorder patients. However, the neural correlates of this therapeutic effect are not yet well-understood. In the current study we investigated whether patients show a strengthening in functional network connectivity in the delta frequency band (1–3.5 Hz) over the course of phase-oriented inpatient trauma treatment while they performed an emotion regulation task. Further, we examined whether neural changes were associated with symptom reduction and improvement in emotion regulation skills.

**Methods:**

Before and after 8 weeks of treatment, electroencephalography (EEG) was acquired in patients (*n* = 28) with a complex posttraumatic stress disorder (cPTSD) or complex dissociative disorder (CDD). They also completed clinical and emotion regulation questionnaires. To delimit data variability, patients participated as one dissociative part that is referred to as Apparently Normal Part (ANP). Patients' data were compared to a matched healthy control croup (*n* = 38), also measured twice.

**Results:**

Prior to treatment, functional connectivity was significantly lower in patients compared to controls during cognitive reappraisal of unpleasant pictures and passive viewing of unpleasant and neutral pictures. These hypoconnected networks largely overlapped with networks typically activated during the recall of (emotional) autobiographical memories. Functional connectivity strength within these networks significantly increased following treatment and was comparable to controls. Patients showed symptom reduction across various clinical domains and improvement in the use of cognitive reappraisal as emotion regulation strategy. Treatment-related network normalizations were not related to changes in questionnaire data.

**Conclusion:**

Phase-oriented treatment may strengthen connections between regions that are activated during autobiographical recall. These findings encourage further investigation of this circuitry as a therapeutic target in cPTSD and CDD patients.

**Clinial trial registration:**

www.ClinicalTrials.gov, identifier: NCT02459340, https://www.kofam.ch/de/studienportal/suche/149284/studie/26681.

## Introduction

According to the Theory of Structural Dissociation of the Personality (1–4) trauma-related disorders can be ranged on a continuum from simple posttraumatic stress disorder (PTSD), to complex (cPTSD), to dissociative disorder not otherwise specified example 1 (DDNOS-1), and dissociative identity disorder (DID). Whereas, simple PTSD is typically associated with a single-incident or limited adult trauma, complex trauma disorders such as cPTSD, DDNOS-1, and DID can arise in response to a history of complex interpersonal trauma during childhood. CPTSD has been listed as an official diagnostic category in ICD-11 (5) and is associated with core PTSD symptoms along with additional symptoms related to emotion regulation, self-concept, and interpersonal relationships (6). DDNOS-1 is a mild form of DID and has therefore been termed Partial DID in ICD-11 (5). In the theory of Structural Dissociation of the Personality (1–4), dissociation is defined as a division of the personality into different dissociative ‘parts'. The more severe the trauma-related disorder on the aforementioned continuum is, the more complex is the division among different dissociative parts. Several prototypical dissociative parts are distinguished (2–4). To date, DID patients have been investigated as fragile Emotional Part (EP)^1^ and Apparently Normal Part (ANP) (7–15). Fragile EP is the part that manages defense to potential threat. This part recollects traumatic memories and is prone to reenact these in sensorimotor and affectively charged ways that typically include mammalian defenses to major threat. These phenomena qualify as positive dissociative symptoms inasmuch as one part has them (here, fragile EP) but not another part (e.g., ANP). ANP aims to fulfill daily life goals, and in this context, it mentally and behaviorally avoids the traumatic past. As a result, ANP tends to have negative dissociative symptoms such as emotional and bodily detachment, depersonalization, derealization, and more or less extensive amnesia for trauma memories (2–4). Consistent with the theory of Structural Dissociation of the Personality, fragile EP and ANP in DID patients show distinct brain and autonomic response patterns in reaction to threat cues (7–9, 13). Fragile EP's reaction pattern includes vegetative hyperarousal and less prefrontal and anterior cingulate activation than ANP and controls. This effect can be interpreted as lack of inhibition on emotional networks including the amygdala and insula. In contrast, ANPs react with vegetative hypoarousal and emotional and bodily detachment that is linked with an excessive prefrontal and anterior cingulate control on emotional networks (11). Because of these findings, it is important to check in which dissociative part(s) traumatized and dissociative patients are measured to prevent mixing different reaction patterns to emotional cues (3).

Exposure therapy is effective in PTSD (16), however, as a stand-alone approach it can cause large side-effects in complex trauma-related disorders (17). The standard care for complex trauma and dissociative disorder patients such as cPTSD, DDNOS-1, and DID is a phase-oriented treatment (18–24). This treatment approach consists of three phases: 1) establishing safety, stabilization, and symptom reduction, 2) treatment of trauma memories, and 3) rehabilitation and personal growth. In the stabilization phase, emotion regulation strategies are taught to ensure that patients can better tolerate trauma exposure. The three phases can be regarded as a recursive spiral where patients can return to previous phases according to their treatment needs (18). A recent meta-analysis showed that the phase-oriented treatment approach is beneficial in reducing PTSD symptoms in cPTSD and dissociative disorder patients (25). Further, highly dissociative individuals show significant improvement when their trauma-related dissociation is addressed in a phase-oriented treatment format [for a review, see (26)].

The neural correlates associated with successful treatment of complex trauma and dissociative disorder patients have hardly been investigated so far. To our knowledge, our previous study (27) is the only study to date that addressed this topic and explored functional connectivity alterations in networks activated during an emotion regulation task across a phase-oriented treatment in complex trauma-related disorders. Patients participating in the study either fulfilled the diagnostic criteria of cPTSD, DDNOS-1, or DID. They received trauma- and dissociation-adapted treatment in a multimodal inpatient setting during 8 weeks. Data were acquired pre- and post-treatment and electroencephalography (EEG) functional connectivity on the source level in the theta (4–8 Hz), alpha (8.5–12 Hz), and beta (12.5–30 Hz) frequency band was assessed. During data acquisition, participants had to either cognitively reappraise unpleasant pictures or to view neutral or unpleasant pictures. Compared to mentally healthy controls, patients showed a pre-treatment hypo-connectivity in networks that disappeared after treatment. These functional connectivity alterations were restricted to the beta frequency band and mainly encompassed cognitive control regions (prefrontal cortex [PFC], anterior cingulate cortex [ACC]), lateral and mesial temporal regions (temporal gyrus, hippocampus/para-hippocampal gyrus), and the insula. In addition, patients showed a pre- to post-treatment reduction in self-reports on PTSD, depression, and general dissociative and negative dissociative symptoms involving depersonalization, derealization, and various anesthetic symptoms. Further, they exhibited a treatment-related increase in the use of cognitive reappraisal that is known to be an effective emotion regulation strategy (28–32). Network changes in the beta frequency band were neither significantly correlated with clinical symptom reduction nor with improved self-reported emotion regulation skills.

Studies relating EEG oscillations with cognitive and emotional processes have mainly focused on theta, alpha, beta, and gamma frequency bands. Some studies addressed slow delta oscillatory activity (1–3.5 Hz). Knyazev and colleagues suggest that delta oscillations modulate activity in brain circuits that are involved in basic motivational processes. These processes facilitate survival by screening of external and internal cues that indicate threat or reward (33, 34). This research implies a key role for delta oscillations in motivation, attention, and salience detection. In line with this interpretation, maximal delta oscillatory response was observed upon presentation of salient and highly arousing cues (35, 36) or during conditions that demand to internally direct the attention when a mental task is executed (e.g., solving an arithmetic problem or working memory task) (37, 38). In a condition where neutral or affectively salient stimuli are presented and participants have to respond naturally or change emotional responses to these cues, a modulation of neural activity in the delta frequency band is expected. Therefore, we here specifically focused on delta oscillations to further explore treatment-related functional connectivity changes and extended previously applied analyses to delta oscillations. All patients were exclusively tested as ANP to reduce variance in the collected data. In accordance with our previous EEG emotion regulation study (27), we hypothesized that phase-oriented treatment strengthens functional connectivity between the PFC, ACC, lateral and mesial temporal areas, and the insula in the patient group. As we did not find any significant correlation between pre- to post-treatment functional connectivity increases and self-reported symptom reduction and/or enhancement in emotion regulation in our precedent article (27), we did not propose any hypotheses on possible correlations between questionnaire data and neural connectivity in the delta frequency band.

## Materials and methods

The current study is part of a larger project designed to investigate treatment-related changes in complex trauma and dissociative disorder patients. The methods applied here largely overlap with the methods used in Schlumpf et al. (27).

### Treatment setting

At the time of measurement, the patients were inpatients on two specialized trauma wards at the Psychiatric Hospital Clienia Littenheid AG, Littenheid, Switzerland that usually lasts 8 weeks. Both wards offer a multimodal phase-oriented program including trauma- and dissociation-specific psychotherapy (in individual and group setting), stabilization groups (cognitive and body-related), and other non-verbal treatment settings (occupational, art, and music therapy). Supplementary Table 1 outlines the treatment modalities applied per patient. Most patients enrolled in the study was in the first and/or second treatment phase.

### Subjects

Data of 21 patients with a cPTSD and 23 with a complex dissociative disorder (CDD) were acquired. CDD patients fulfilled criteria of a DDNOS-1 or DID (39). The Structural Diagnostic Interview for DSM-IV for Dissociative Disorders (SCID-D) (40) and the Posttraumatic Diagnostic Scale (PDS) (41) were used to verify the clinical diagnoses of DID, DDNOS-1, and PTSD. As cPTSD has become an official diagnose in ICD-11 (5) after the completion of data acquisition, the consensus criteria of cPTSD were checked using the German Version of the Structured Interview for Disorders of Extreme Stress, that is, the *Interview zur Komplexen Posttraumatischen Belastungsstörung* (IK-PTBS) (42). All patients suffered from chronic and severe interpersonal trauma. To delimit data variability, patients were exclusively measured as ANP. The reason for choosing ANP is that it is easier to control than EP in an experimental setting. Further, most patients were not able to willfully bring forward an EP and to stay in this part throughout the experiment. The control group consisted of 40 in age and sex matched healthy controls. Due to various reasons, we had several drop-outs: premature discharge from the clinic (2 cPTSD), inability to perform the experimental task (4 cPTSD, 4 CDD), low number of artefact-free segments (see below; 1 cPTSD, 1 CDD, 1 healthy control), technical problems during data acquisition (4 cPTSD), or back up error (1 healthy control). The final analysis was conducted in 18 CDD patients, 10 cPTSD patients, and 38 healthy controls. The reader is referred to Table 1 for details on demographic and clinical characteristics of participants and Supplementary Table 2 for details on psychotropic medication and comorbid diagnoses in the patient group (27).

**Table 1:** Demographic and clinical data.

	**Patients (*****n*** = **28)**			**Controls (*****n*** = **38)**			
**Demographic measures**							***p*-value (patients vs. controls)**
Sex	22 female / 6 male			31 female / 7 male			n.a.
Education	High school: 75%, college: 25%			High school: 50%, college: 50%			n.a.
Age	42.04 (10.18)			41.37 (12.71)			0.81
Days between pre to post	40.90 (1.29)			49.00 (1.25)			<0.0000
**Clinical measures**	**Pre**	**Post**	***p*** **-value (** ***post-hoc t*** **-test)**	**Pre**	**Post**	***p*** **-value (** ***post-hoc t*** **-test)**	***p*** **-value (main effect of group)**
PCL-C total	56.40 (10.45)	50.83 (10.94)	<0.0001	19.44 (4.02)	19.24 (4.16)	0.89	<0.0001
FDS	24.17 (14.51)	20.66 (12.85)	0.002	2.30 (2.15)	1.65 (1.77)	0.54	<0.0001
SDQ-20	35.26 (9.79)	33.43 (10.18)	-	20.66 (1.32)	20.66 (1.55)	-	<0.0001
PosDiss	15.00 (10.34)	13.55 (9.14)	-	1.64 (1.67)	1.35 (1.43)	-	<0.0001
NegDiss	20.76 (10.89)	17.08 (9.91)	0.0005	2.67 (2.13)	1.93 (1.62)	0.42	<0.0001
BDI-II	28.30 (11.16)	22.90 (10.15)	0.001	2.27 (4.71)	1.78 (3.24)	0.61	<0.0001
STAI-T	55.02 (8.40)	54.07 (7.10)	-	27.82 (6.63)	27.16 (7.71)	-	<0.0001
DERS total	108.35 (23.86)	104.57 (23.82)	-	55.00 (16.40)	53.63 (14.00)	-	<0.0001
ERQ_Reappraisal	20.85 (7.61)	24.85 (6.36)	108.35	30.82 (6.37)	31.18 (5.41)	0.69	<0.0001
ERQ_Suppression	17.44 (5.58)	17.89 (4.51)	-	11.57 (4.78)	11.00 (4.44)	-	<0.0001

*M, mean; SD, standard deviation; N.a., not applicable; pre, pre-treatment; post, post-treatment; PCL-C, Post-traumatic Stress Disorder Checklist, civilian version; FDS, Fragebogen zu Dissoziativen Symptomen; SDQ-20, Somatoform Dissociation Questionnaire; BDI-II, Beck's Depression Inventory; STAI-T, Stait-Trait Anxiety Inventory; DERS, Difficulty in Emotion Regulation Scale; ERQ, Emotion Regulation Questionnaire. P-values are two-sided and FDR corrected for post-hoc t-tests. Post-hoc t-tests were only performed if the interaction effect (group x time point) was significant. Effect sizes were calculated as generalized eta^2^ for main and interaction effects and as Cohen's d for t-tests*.

Written informed consent from each human subject was obtained prior to his/her participation. The study was approved by the local ethics committees of the cantons Zurich and Thurgau. All procedures performed in this study were in accordance with the Declaration of Helsinki.

### Study design

We acquired data at two time points. The patient group was tested pre-treatment (at the beginning of their inpatient stay) and post-treatment (before discharge from the hospital). Controls were examined twice as well-within a time period of 5 to 10 weeks. Each data acquisition included an EEG experiment and the assessment of self-reports on clinical symptoms and emotion regulation capacity.

#### EEG paradigm

**Figure 1:**
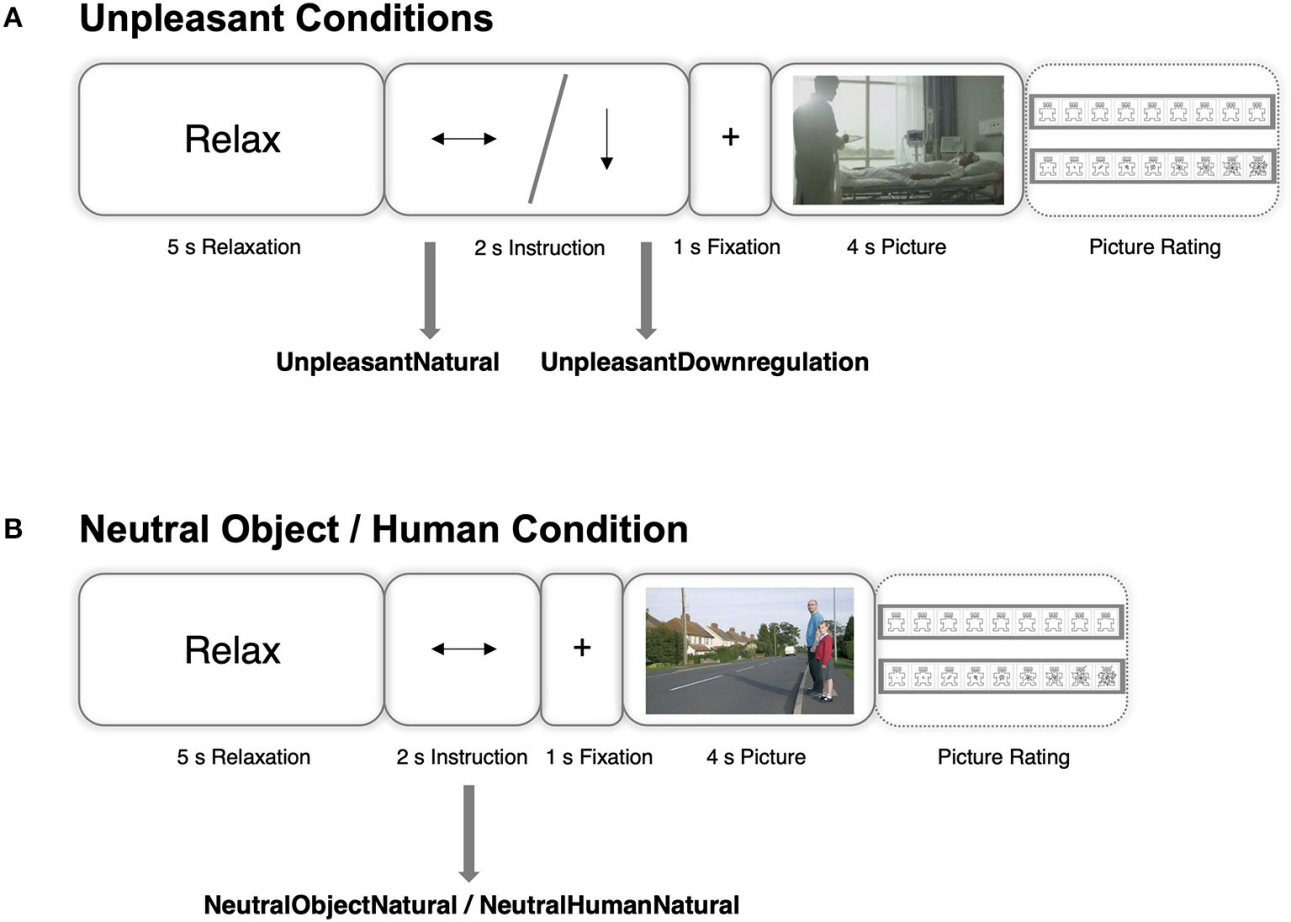
Schematic representation of an example trial in each condition. A horizontal arrow or a vertical arrow pointing downwards indicate that the individual has to naturally respond to or to reduce the emotional reaction to an upcoming picture, respectively. **(A)** Depicts trials that use unpleasant images, **(B)** depicts trials that use neutral object or neutral human pictures. From “Functional reorganization of neural networks involved in emotion regulation following trauma therapy for complex trauma disorders” by Schlumpf et al. (27). CC BY-NC-ND 4.0.

The EEG task comprised a cognitive reappraisal task that was developed according to previous cognitive reappraisal EEG studies (43–47). Cognitive reappraisal is a strategy that aims to re-interpret a stressful situation in a way to change its perceived emotional impact (48). Prior to the experiment, participants were instructed how to use self- or situation-focused cognitive reappraisal strategies [according to (49)]. Situation-focused reappraisal refers to the re-interpretation of situational aspects of a situation (e.g., imagining that the crying person on the picture will get better soon). Self-focused reappraisal refers to the re-evaluation of the self-relevance of a situation by taking a detached, third-person perspective (e.g., imagining that the picture depicts a movie rather than a real incident). Color pictures were taken from the International Affective Picture System (IAPS) (50). The following types of pictures were presented: 1) highly arousing pictures depicting humiliation, threat, and grief (unpleasant); 2) neutral pictures depicting plants, landscapes, and household objectives (neutral object); 3) neutral pictures comprising neutral interpersonal scenes or neutral faces [neutral human; for more details on the picture selection process, see supplementary materials in Schlumpf et al. (27)]. A trial is presented in Figure 1. For unpleasant pictures, participants were either requested to have their natural emotional responses to a following picture (UnpleasantNatural condition) or to reduce emotional arousal using cognitive reappraisal (UnpleasantDownregulation condition, see Figure 1A). In trials depicting either neutral objects or neutral human pictures, participants were only instructed to respond naturally to the upcoming picture (NeutralObjectNatural and NeutralHumanNatural condition, see Figure 1B). Each condition was presented 20 times in randomized order. In half of the trials, participants had to rate the pictures regarding valence and arousal using the 9-point Self-Assessment Manikin scale (51). The EEG recording lasted approximately 15 min per measurement point. Results on valence and arousal ratings are outlined in Figure 2 in Schlumpf et al. (27). These ratings suggested that patients perceived all pictures at both time points as more negative and more arousing compared to controls. Further, not only unpleasant but also neutral human pictures evoked abnormal emotional arousal in the patient group.

#### Self-report instruments: Clinical symptoms and emotion regulation capacity

At each measurement point, participants completed several self-report instruments. The civilian version of the Posttraumatic Stress Disorder Checklist (PCL-C) (52) is a 17-items questionnaire that evaluates the severity of DSM-IV PTSD symptom criteria. The items are scored on a scale from 1 to 5 (range: 17–85). The total score measures hyperarousal, intrusion, and avoidance/numbing. The *Fragebogen zu Dissoziativen Symptomen* (FDS) (53, 54) consists of 44 items that evaluate the severity of cognitive-emotional and several other dissociative symptoms. Participants have to indicate the amount of time (0–100%) they experience each symptom. The total score is calculated by summing up the 44 items score and dividing by 44 (range: 0–100). The Somatoform Dissociation Questionnaire (SDQ) (55) assesses the severity of somatoform (i.e., sensorimotor) dissociative symptoms. This questionnaire consists of 20 items that are scored on a scale from 1 and 5 (range: 20–100). Positive dissociative symptoms (e.g., intrusions, flashbacks) and negative dissociative symptoms (e.g., depersonalization, derealization, various anesthetic symptoms) were measured by composite scores including items of the FDS and SDQ-20 that were unambiguously assignable as positive (PosDiss score) or negative (NegDiss score), respectively [see (27)]. The Beck's-Depression Inventory II (BDI-II) comprises 21 items measuring the severity of depression (56). The items are scored on a scale from 0 to 3 (range: 0–63). The trait part of the Stait-Trait Anxiety Inventory (STAI-T) was used to assess trait anxiety (57). This questionnaire consists of 20 items that are scored on a scale from 1 to 4 (range: 20–80).

The Difficulty in Emotion Regulation Scale (DERS) (58) and the Emotion Regulation Questionnaire (ERQ) (59) were assessed at both time points to test emotion regulation strategies. The DERS consists of 36 items that are scored on a scale from 1 to 5 (range 36–180). The DERS total score comprises six subscales (non-acceptance of emotional responses, difficulty in goal-directed behavior, difficulty controlling impulses, lack of emotional awareness, lack of access to emotion regulation strategies, lack of emotional clarity). Higher scores suggest more severe emotion regulation difficulties. The ERQ encompasses the two subscales cognitive reappraisal (ERQ_Reappraisal) and expressive suppression (ERQ_Suppression). The 10 items are scored on a scale from 1 to 7 (range per subscale: 5–35). The higher a subscale score, the more an individual applies the corresponding strategy. Handling of incomplete data is explained in the supplementary material of Schlumpf et al. (27).

We calculated internal consistency (Cronbach's alpha) for each scale and for both measurement points separately. All values are high and are listed in Supplementary Table 3.

### EEG recording and raw data pre-processing

EEG data were registered using an actiCap system in combination with a QuickAmp-72 amplifier (Brain Products Inc., http://www.brainproducts.com). Sixty-four channels were attached according to the international 10–10 electrode placement system. The average of activity at all electrodes was taken and used as a reference. During EEG recording, data were sampled at 500 Hz and filtered with a band-pass filter between 0.1 and 100 Hz and a notch filter at 50 Hz. Impedances were kept below 25 kOhm.

Preprocessing of the raw EEG data was performed using the Brain Vision Analyzer 2.0 software (Brain Products Inc.). Independent component analysis was applied to remove eye activity artifacts (i.e., saccades and eye blinks) (60). Data was then band-pass filtered between 0.1 and 40 Hz. Bad channels were reconstructed based on the interpolated values from the surrounding electrodes. Remaining artifacts (i.e., movement or muscle artifacts) were rejected using the automated raw data inspection implemented in Brain Vision Analyzer. Data were segmented into epochs of 4 s consisting of the data acquired during picture presentation. Using an acceptance criterion of 10 or more artefact-free segments per condition, three participants (1 cPTSD, 1 CDD, 1 healthy control) were excluded from the analysis due to low data quality. Thus, the artefact-free data epochs in all participants ranged from 11 to 20. Further details on data epochs can be found in the supplementary material of Schlumpf et al. (27).

### Connectivity analysis on the source level

The artefact-free and segmented data was exported to the sLORETA toolbox (Version 20160611, https://www.uzh.ch/keyinst/loreta.htm) for further analyses (61). Intracranial functional connectivity values were calculated between 84 regions of interest (ROIs). These 84 ROIs relate to Brodmann areas implemented in sLORETA (BA; 42 for each hemisphere). The labels of brain regions are based on visual inspection and the Juelich Histological and the Harvard-Oxford cortical atlases that are integrated in the fMRIB software (http://fsl.fmrib.ox.ac.uk/fsl/fslwiki/Atlases). The labels and coordinates of all 84 ROIs are listed in Supplementary Table 4. Details on quality check of the source estimation process are provided in the supplementary material of Schlumpf et al. (27). We used lagged coherence as functional connectivity measure. This measure is, compared to the instantaneous coherence value, not confounded by non-physiological artifacts such as low spatial resolution or volume conduction (62, 63). The 4 s preprocessed segments were divided into 2 s segments to increase statistical power. Lagged coherence values in the delta frequency band (1–3.5 Hz) were calculated between the centroid voxel of all pairs of 84 ROIs. Discrete Fourier transform was used to derive the spectral representation of the EEG signal.

### Network-based statistical analyses

Based on these 84 × 84 connectivity matrices from the intracranial analysis in sLORETA, we run network-based statistics using the Network-based Statistic toolbox (NBS, https://www.nitrc.org/projects/nbs/) in MATLAB (version R2015b, http://www.mathworks.com/). This method aims to identify brain graphs that consist of brain regions (nodes) and connections between these areas (edges). Foremost, a sensitivity threshold has to be predefined. Then, a statistical test (e.g., *t*-test) is conducted on every single edge of the network. Edges that exceed the predefined sensitivity threshold form a graph. To make inference, permutation is used and the family-wise error (FWE) rate is controlled by running mass-univariate testing on all graph edges. The statistical significance of a graph expresses the likelihood of finding a graph with an equal or a greater number of edges by chance (64, 65).

To increase statistical power, CDD and cPTSD patients were merged to one group (*n* = 28)^2^. We followed the same statistical approach as in Schlumpf et al. (27). First, between-group differences were examined at the first measurement point for each condition (NeutralObjectNatural, NeutralHumanNatural, UnpleasantNatural, and UnpleasantDownregulation condition) separately. These four two-sample *t*-tests were performed for both contrasts (patients > controls, controls > patients). Second, we evaluated group x time point interactions. These interaction effects were calculated using difference maps (i.e., lagged coherence post-treatment values – lagged coherence pre-treatment values) that were submitted to two-sample *t*-tests. The difference maps were restricted to the networks that significantly differed between groups at the first measurement point. This approach enabled us to check for any treatment related alterations in these networks. Further, we performed two-sample *t*-tests to assess any post-treatment group differences in the initially altered networks. Finally, we also checked for any whole brain post-treatment group differences to explore potential network changes irrespective of the pre-treatment group differences. *P*-values were set to 0.05 and 5,000 permutations were used for all statistical tests. The number of days between measurement points differed significantly between groups (patients M = 40.90 (1.29), controls M = 49.00 (1.25); *t* = −4.51, *p* < 0.000, d = −1.10). Therefore, days between measurements were entered as covariate of no interest in the NBS analyses.

For the pre-treatment analyses, we chose the highest (i.e., most conservative) sensitivity thresholds reaching a significant single subnetwork (NeutralObjectNatural threshold at *t* = 4.0, NeutralHumanNatural threshold at *t* = 3.6, UnpleasantNatural threshold at *t* = 3.8, UnpleasantDownregulation threshold at *t* = 3.2). Thus, we did not select subnetworks that fall apart in different components when using a higher threshold. To ensure that we present stable findings, the pre-treatment analyses had to reveal significant results at least for a range of three thresholds when descending them in 0.1 steps. The most liberal sensitivity threshold displaying a significant result was chosen for group x time point interaction and post-treatment group analyses. This approach ensured to fully examine if initially altered networks changed across time.

The BrainNet Viewer was used to visualize the functional brain networks (www.nitrc.org/projects/bnv/) (66). Cohen's d, mean, and standard deviations were specified for each significant network revealed in the NBS analysis. These values were computed in R (version 3.4.0, https://www.r-project.org) based on mean functional connectivity scores of these networks. A mean value was calculated by averaging the coherence values of all edges of a network.

### Relationship between functional connectivity and self-reports

We tested whether treatment-related changes of the patients' functional connectivity strength were associated with alterations in clinical symptoms and emotion regulation capacity. For this purpose, mean functional connectivity values per network revealed by the group x time point NBS analyses were calculated. Changes across treatment in self-report instruments were investigated by subtracting the pre-treatment score of each patient in a questionnaire from the associated post-treatment score. These difference values (Diff_PCL-C, Diff_FDS, Diff_SDQ-20, Diff_PosDiss, Diff_NegDiss, Diff_BDI-II, Diff_STAI_T, Diff_DERS, Diff_ERQ_Reapraisal, Diff_ERQ_Suppression) were correlated with the mean functional connectivity values per network using Spearman's rank correlations. Correlational analyses were performed in R as two-tailed tests and were limited to the patient's group only. We used false discovery rate (FDR) to correct for multiple comparisons (67). FDR adjustment was applied separately for self-reports on clinical symptoms and self-reports on emotion regulation capacity.

### Treatment-related changes in self-report instruments

For each self-report instrument, we performed two (groups) x two (time points) mixed-design ANOVAS. These statistical tests were conducted in R. We used the afex package (68) for factorial designs and applied a Greenhouse-Geisser correction to within subject factors if the assumption of sphericity was violated. *P*-values are two-tailed. In *post-hoc t*-tests, we applied FDR correction (67) to adjust for multiple comparisons. Effect sizes are reported as Cohen's d (69) for *t*-tests and as generalized eta^2^ (70) for main and interaction effects.

## Results

In the present study, we investigated functional connectivity changes in emotion regulation networks induced by a phase-oriented inpatient treatment setting in patients with a history of chronic and severe interpersonal trauma. We extended our analyses of Schlumpf et al. (27) to delta oscillatory responses as they have been shown to be involved in the processing of salient and highly arousing cues (35, 36) or during conditions that demand internally directed attention (36, 37).

### Network-based statistics

Pre-treatment and compared to the control group, patients showed hypoconnected networks in the delta frequency band in all conditions [NeutralObjectNatural: *p* = 0.011, FWE corrected, Cohen's d = −0.80, NBS-specific threshold at *t* = 4.0, patients mean (SD): 0.05 (0.03), controls mean (SD): 0.08 (0.04); NeutralHumanNatural: *p* = 0.015, FWE corrected, Cohen's d = −1.10, NBS-specific threshold at *t* = 3.6, patients mean (SD): 0.07 (0.03), controls mean (SD): 0.11 (0.05); UnpleasantNatural: *p* = 0.025, FWE corrected, Cohen's d = −1.07, NBS-specific threshold at *t* = 3.8, patients mean (SD): 0.05 (0.02), controls mean (SD): 0.09 (0.04); UnpleasantDownregulation: *p* = 0.042, FWE corrected, Cohen's d = −0.88, NBS-specific threshold at *t* = 3.2, patients mean (SD): 0.08 (0.04), controls mean (SD): 0.14 (0.08)]. The network in the NeutralObjectNatural condition comprised four left lateralized nodes and three edges involving the frontal pole, inferior temporal gyrus, and parahippocampal gyrus. In the NeutralHumanNatural condition, the network consisted of eight left lateralized nodes and eight edges encompassing the ventrolateral prefrontal cortex (vlPFC), posterior cingulate cortex (PCC), superior parietal lobule, superior temporal gyrus, parahippocampal gyrus, hippocampus, and insula. Regarding the UnpleasantNatural condition, the analysis revealed three hypoconnected edges between four left lateralized nodes encompassing the vlPFC, superior parietal lobule, superior temporal gyrus, and insula. In the UnpleasantDownregulation condition, the pre-treatment hypoconnected network involved eight nodes and nine edges between left to right connections. This network comprised of the PCC, precuneus, cuneus, occipital pole, and lingual gyrus.

**Figure 2:**
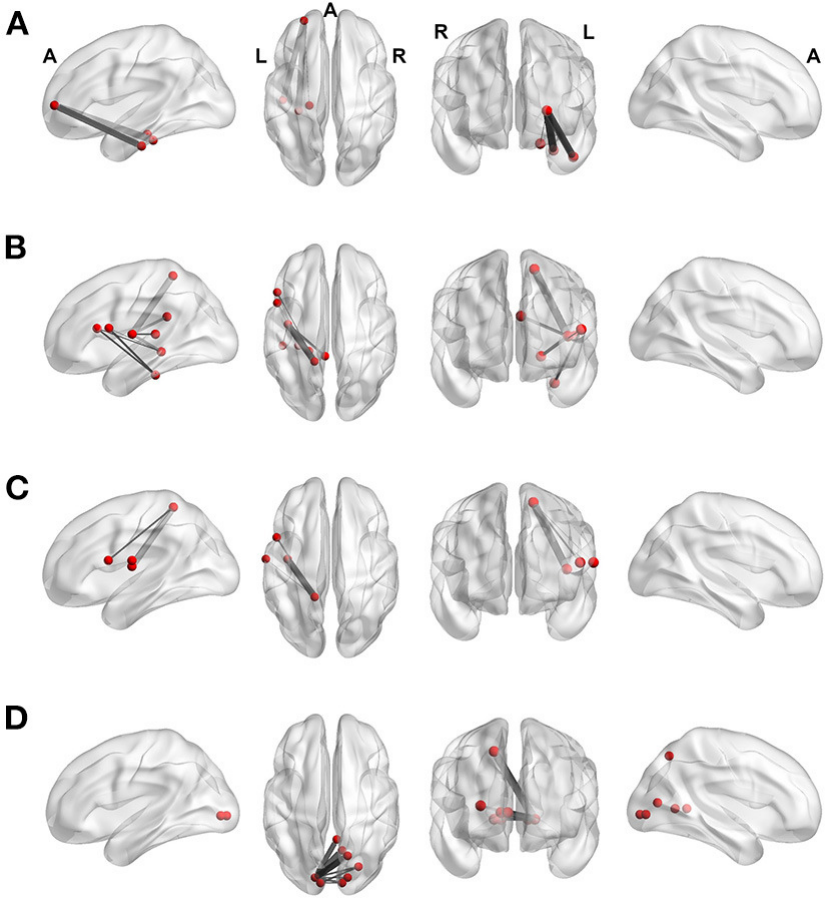
Functional connectivity increase in the delta frequency band over the course of treatment within the initially reduced network in the patient group (group x time point interaction) in the **(A)** NeutralObjectNatural, **(B)** NeutralHumanNatural, **(C)** UnpleasantNatural, and **(D)** UnpleasantDownregulation condition. Red dots display nodes, the gray lines correspond to the connections (edges). The thickness of a line expresses the significance (*t*-value) of a connection (*p* < 0.05, FWE corrected). Inter- and intrahemispheric connections are shown in left, right, horizontal, and coronal slices. A, anterior; L, left; R, right.

Second, we investigated whether functional connectivity changed from the first to the second measurement within these networks. Group x time point interactions revealed a significant pre- to post-treatment functional connectivity increase in the patient group. The patients' priorly hypoconnected networks had disappeared in all conditions after treatment [NeutralObjectNatural: *p* = 0.04, FWE corrected, Cohen's d = 0.41, NBS-specific threshold at *t* = 1.3, patients mean (SD): 0.01 (0.05), controls mean (SD): −0.01 (0.05); NeutralHumanNatural: *p* = 0.05, FWE corrected, Cohen's d = 0.71, NBS-specific threshold at *t* = 0.4, patients mean (SD): 0.02 (0.05), controls mean (SD): −0.02 (0.06); UnpleasantNatural: *p* = 0.05, FWE corrected, Cohen's d = 0.66, NBS-specific threshold at *t* = 1.0, patients mean (SD): 0.01 (0.04), controls mean (SD): −0.02 (0.05); UnpleasantDownregulation: *p* = 0.05, FWE corrected, Cohen's d = 0.35, NBS-specific threshold at *t* = 0.6, patients mean (SD): 0.02 (0.06), controls mean (SD): 0.00 (0.10)]. These networks are depicted in Figure 2. Tables 2–5 list the edges and nodes involved in these networks. Functional connectivity changes (mean of lagged coherence values) per group and individual pre- to post-treatment trajectories are depicted in Supplementary Figures 1, 2, respectively.

**Table 2:** Patients' functional connectivity increase across treatment within the initially impaired network in the NeutralObjectNatural condition.

**Node**				**Node**						
**BA**	**L/R**	**MNI coordinates (x, y, z)**	**Brain region**	**BA**	**L/R**	**MNI coordinates (x, y, z)**	**Brain region**	***t-*value**	**Diff patients**	**Diff controls**
10	L	(−25, 55, 5)	Frontal pole	36	L	(−30, −30,−25)	Parahippocampal gyrus	1.94	0.010	−0.012
10	L	(−25, 55, 5)	Frontal pole	20	L	(−45, −20, −30)	Inferior temporal gyrus (Fusiform gyrus)	1.92	0.014	−0.006
10	L	(−25, 55, 5)	Frontal pole	35	L	(−20, −25, −20)	Parahippocampal gyrus	1.65	0.008	−0.011

*BA, Brodmann area; L, left hemisphere; R, right hemisphere; MNI, Montreal Neurological Institute; Diff patients, difference of mean lagged coherence value in the patients (post-treatment value – pre-treatment value); Diff controls, difference of mean lagged coherence value in controls (post-treatment value – pre-treatment value). In the sLORETA toolbox, several BAs have two centroid voxels (specified with a and b). NBS-specific threshold at t = 1.3, p < 0.05 (FWE corrected)*.

**Table 3:** Patients' functional connectivity increase across treatment within the initially impaired network in the NeutralHumanNatural condition.

**Node**				**Node**						
**BA**	**L/R**	**MNI coordinates (x, y, z)**	**Brain region**	**BA**	**L/R**	**MNI coordinates (x, y, z)**	**Brain region**	***t*-value**	**Diff patients**	**Diff controls**
5	L	(−15, −45, 60)	Superior parietal lobule	13	L	(−40, −10, 10)	Insular cortex	3.75	0.025	−0.014
13	L	(−40, −10, 10)	Insular cortex	27	L	(−20, −35, −5)	Hippocampus	2.59	0.025	−0.019
13	L	(−40, −10, 10)	Insular cortex	23	L	(−5, −40, 25)	Cingulate gyrus (PCC)	2.53	0.017	−0.011
36	L	(−30, −30, −25)	Parahippocampal gyrus	44	L	(−50, 10, 15)	Inferior frontal gyrus (Pars opercularis, vlPFC)	2.38	0.014	−0.028
36	L	(−30, −30, −25)	Parahippocampal gyrus	45	L	(−50, 20, 15)	Inferior frontal gyrus (Pars triangularis, vlPFC)	2.23	0.009	−0.031
13	L	(−40, −10, 10)	Insular cortex	41a	L	(−55, −25, 5)	Superior temporal gyrus	2.14	0.029	−0.003
27	L	(−20, −35, −5)	Hippocampus	45	L	(−50, 20, 15)	Inferior frontal gyrus (Pars triangularis, vlPFC)	2.02	0.010	−0.028
27	L	(−20, −35, −5)	Hippocampus	44	L	(−50, 10, 15)	Inferior frontal gyrus (Pars opercularis, vlPFC)	1.85	0.006	−0.023

*BA, Brodmann area; L, left hemisphere; R, right hemisphere; MNI, Montreal Neurological Institute; Diff patients, difference of mean lagged coherence value in the patients (post-treatment value – pre-treatment value); Diff controls, difference of mean lagged coherence value in controls (post-treatment value – pre-treatment value); vlPFC, ventrolateral prefrontal cortex; PCC, posterior cingulate cortex. In the sLORETA toolbox, several BAs have two centroid voxels (specified with a and b). NBS-specific threshold at t = 0.4, p < 0.05 (FWE corrected)*.

**Table 4:** Patients' functional connectivity increase across treatment within the initially impaired network in the UnpleasantNatural condition.

**Node**				**Node**						
**BA**	**L/R**	**MNI coordinates (x, y, z)**	**Brain region**	**BA**	**L/R**	**MNI coordinates (x, y, z)**	**Brain region**	***t*-value**	**Diff patients**	**Diff controls**
5	L	(−15, −45, 60)	Superior parietal lobule	13	L	(−40, −10, 10)	Insular cortex	2.86	0.019	−0.024
5	L	(−15, −45, 60)	Superior parietal lobule	44	L	(−50, 10, 15)	Inferior frontal gyrus (Pars opercularis, vlPFC)	2.1	0.007	−0.015
5	L	(−15, −45, 60)	Superior parietal lobule	42b	L	(−60, −10, 15)	Superior temporal gyrus	1.9	0.016	−0.010

*BA, Brodmann area; L, left hemisphere; R, right hemisphere; MNI, Montreal Neurological Institute; Diff patients, difference of mean lagged coherence value in the patients (post-treatment value – pre-treatment value); Diff controls, difference of mean lagged coherence value in controls (post-treatment value – pre-treatment value); vlPFC, ventrolateral prefrontal cortex. In the sLORETA toolbox, several BAs have two centroid voxels (specified with a and b). NBS-specific threshold at t = 1.0, p < 0.05 (FWE corrected)*.

**Table 5:** Patients' functional connectivity increase across treatment within the initially impaired network in the UnpleasantDownregulation condition.

**Node**				**Node**						
**BA**	**L/R**	**MNI coordinates (x,y,z)**	**Brain region**	**BA**	**L/R**	**MNI coordinates (x,y,z)**	**Brain region**	***t*-value**	**Diff patients**	**Diff controls**
17b	L	(−15, −85, 0)	Lingual gyrus	7	R	(15, −65, 50)	Precuneus	2.91	0.018	−0.022
17b	L	(−15, −85, 0)	Lingual gyrus	30b	R	(10, −60, 5)	Cuneus	2.89	0.045	0.000
17b	L	(−15, −85, 0)	Lingual gyrus	29	R	(5, −50, 5)	Cingulate gyrus (PCC)	2.56	0.039	0.001
17b	L	(−15, −85, 0)	Lingual gyrus	17a	R	(10, −90, 0)	Lingual gyrus	1.73	0.022	0.000
17b	L	(−15, −85, 0)	Lingual gyrus	30a	R	(25, −75, 10)	Cuneus	1.66	0.017	0.000
17a	L	(−10, −90, 0)	Occipital pole (Primary visual cortex)	30a	R	(25, −75, 10)	Cuneus	1.65	0.018	−0.001
17a	L	(−10, −90, 0)	Occipital pole (Primary visual cortex)	17a	R	(10, −90, 0)	Occipital pole (Primary visual cortex)	1.49	0.017	−0.003
17b	L	(−15, −85, 0)	Lingual gyrus	17b	R	(15, −85, 0)	Lingual gyrus	1.43	0.020	0.006
17a	L	(−10, −90, 0)	Occipital pole (Primary visual cortex)	17b	R	(15, −85, 0)	Lingual gyrus	1.23	0.014	0.003

*BA, Brodmann area; L, left hemisphere; R, right hemisphere; MNI, Montreal Neurological Institute; Diff patients, difference of mean lagged coherence value in the patients (post-treatment value – pre-treatment value); Diff controls, difference of mean lagged coherence value in controls (post-treatment value – pre-treatment value); PCC, posterior cingulate cortex. In the sLORETA toolbox, several BAs have two centroid voxels (specified with a and b). NBS-specific threshold at t = 0.6, p < 0.05 (FWE corrected)*.

Third, we checked for post-treatment group differences in the initially hypoconnected networks and did not find any significant network differences (*p* > 0.05). Thus, patients' pre-treatment hypoconnected networks had normalized post-treatment in all conditions. Finally, we compared the groups at the second time point on the whole-brain level. There were no significant group differences regarding any of the experimental conditions (all *ps* > 0.05).

### Correlations between functional connectivity and self-reports across treatment

We calculated correlations between treatment-related network changes and changes in self-reports on clinical symptoms and emotion regulation capacity. No Spearman's rank correlation survived FDR correction (all *ps* > 0.05).

### Treatment-related changes in clinical symptoms and emotion regulation capacity

Two (groups) x two (time points) mixed-design ANOVAs were performed for each self-report instrument on clinical symptoms and emotion regulation capacity. Across all measures and both measurement points, we revealed more clinical symptoms and more severe emotion regulation deficits in patients compared to controls. Patients exhibited a significant symptom reduction from pre- to post-treatment in the patient group in overall PTSD symptoms (PCL-C total), general dissociative symptoms (FDS), negative dissociative symptoms, and depression (BDI-II). Further, we revealed a significant treatment-related increase in reappraisal values. Table 1 provides detailed information.

## Discussion

This is the first study to show that neural networks in the delta frequency band in cPTSD, DDNOS-1, and DID patients change following trauma treatment. Pre- and post-treatment, we measured EEG to calculate delta oscillatory functional connectivity at the source-level in networks involved in cognitive reappraisal of unpleasant pictures or viewing neutral and unpleasant pictures. Before treatment, patients showed hypoconnected networks in all experimental conditions in comparison to a healthy control group. Post-treatment, the patients' functional network strength increased to the level of the healthy controls. This increase was not related to an improvement in clinical symptoms or emotion regulation skills.

During passive viewing of neutral and unpleasant pictures (NeutralObjectNatural, NeutralHumanNatural, and NeutralUnpleasant condition), we observed functional network changes from pre- to post-treatment in the patients within prefrontal regions, the PCC, superior parietal lobule, lateral and mesial temporal areas, and the insula. Network alterations were found exclusively in the left hemisphere. These networks largely overlap with a predominantly left lateralized network typically found in autobiographical memory retrieval (71–74).

Treatment-related network changes during cognitive reappraisal of unpleasant pictures (UnpleasantDownregulation condition) encompassed the PCC, precuneus, and several occipital areas (cuneus, occipital pole, lingual gyrus). All these regions have also been implicated in autobiographical memory (71). However, compared to the networks activated in the viewing conditions, this network was not lateralized but encompassed edges in both hemispheres. Bilateral brain activity has been found in several studies investigating emotional memory retrieval (71). Hence, our findings support the idea that treatment brought about increased connectivity of a neural network associated with the retrieval of emotional autobiographical events while the patients were instructed to reappraise aversive cues. We had expected to find prefrontal and anterior cingulate regions in this network as they are typically implicated in cognitive reappraisal (75–77). The lack of these regions suggests that delta oscillatory responses were not associated with group differences in cognitive reappraisal. Both groups perceived the unpleasant pictures after treatment as significantly less negative compared to before treatment. However, the patients' emotional ratings of the unpleasant pictures indicate that these appeared more negative and arousing to them compared to the healthy control group before and after treatment (12). Thus, the aversive pictures continued to have a negative meaning to them which is probably due to their trauma history.

Our results might reflect an increased capacity in patients as ANP to recollect their (painful) past, a past that they somehow associated with the neutral and unpleasant pictures. Clinical and empirical evidence shows that ANPs are typically associated with mental and behavioral avoidance of trauma-related cues (2–4, 7–9, 13–15). Most of the patients under study were in the first and/or second treatment phase that focuses on building up skills to react to emotional challenges in effective ways and on controlled confrontation with traumatic experiences (18–24). A primary goal in these phases for ANPs is to raise the capacity to tolerate strong affect, sensations, and memories related to aversive past events. This is a requirement for the integration of traumatic memories in one's autobiography and fusion of dissociative parts (2–4). To the extent that treatment is effective, ANPs may be expected to be less emotionally and physically numbed not only with regards to the trauma history but far more generally and have gained the ability to mentally avoid their common and traumatizing past less. Consequently, they might be better able to recollect their (painful) past. Hence, our results might reflect a significant treatment progress in the patients under study and is in accordance with the treatment-related decrease in negative dissociative symptoms (see Table 1) that implies that patients as ANP were less depersonalized, de-realized, and emotionally numbed following phase-oriented treatment (12).

In line with our hypothesis, we found a strengthening of neural functional networks across treatment in the patient group. However, we did not expect that these networks would show a large similarity with the autobiographical memory network. Autobiographical memories entail great personal and emotional significance (78, 79). Hence, our findings might be particularly mediated by the functional roles of delta oscillations. Delta band activity is associated with the perception of cues that are motivationally and emotionally salient and attract attention (33–38). In this line of reasoning, a strengthening in the autobiographical memory network in response to visual cues could also reflect that treatment may have helped the patients as ANP to experience and see more what is self-relevant and of affective significance to them. This interpretation is in accordance with a resting-state functional connectivity study suggesting an increased ability in ANPs to engage in self-related thinking across a phase-oriented inpatient treatment (80) and is further supported by the growing body of evidence that relates dissociation to a reduce capacity for self-reference (81–83). The increased functional coupling among occipital areas in the network involved in the execution of cognitive reappraisal suggests as well-that the patients experienced and perceived unpleasant pictures more intensely following treatment while they managed to reduce their emotional responses to these cues.

The interpretation of our results remains speculative as we did not measure participants' mental state during the EEG measurement. In particular, we did not systematically explore if the presented pictures promoted autobiographical retrieval. It is therefore important to investigate in a follow-up study whether the pictures led to a reactivation of associations with past (traumatizing) events. However, our interpretation is in line with the capacities that are gradually developed in a phase-oriented treatment. In the patients' discharge reports, therapists reported improvements regarding containment and grounding strategies, awareness and tolerance of sensations and affects associated with the trauma and other dissociative parts, identification of triggers, remembering painful past events, realization that these are part of their life and acceptance of their meaning and implications, and becoming better reoriented in the actual presence while recollecting their past. These evaluations are consistent with the observed network changes and our interpretation thereof.

Patients experienced a significant therapy-related reduction in PTSD symptoms (PCL-C), general dissociative symptoms (FDS), and negative dissociative symptoms (NegDiss), and symptoms of depression (BDI-II) (see Table 1). Symptom reduction across several clinical domains is in accordance with cross-sectional (84) and longitudinal treatment outcome studies (85, 86). Further, the patients' use of cognitive reappraisal as emotion regulation strategy increased significantly following treatment. Nonetheless, patients still had higher values in all clinical measures after treatment compared to healthy controls. Thus, a continuation of treatment is required. Previous findings of relationships between alterations in questionnaire data and neural changes across treatment are inconsistent (27, 80, 87–90). In the present study, network changes were neither associated with self-reported symptom reduction, nor with improvement in emotion regulation capacities. A reason for the null finding on correlations between neural and questionnaire data might be that network changes in the delta frequency band do not particularly relate to changes in the assessed clinical and behavioral variables. Electrophysiological outcome in the current study might for instance rather be associated with autobiographical recall that has not been tested.

There are several limitations to this study. We did not include a waiting list control group. Nevertheless, due to the chronicity of the patients' pathology, we do not assume that time alone can explain the treatment related network changes. Patients showed high comorbidity and took psychotropic medication. Hence, confounding effects of comorbid disorders and psychotropic medication cannot be excluded. However, the study conditions were naturalistic as the patients enrolled in the study typically show high comorbidity and take psychopharmaca. Further, since individual medical therapies were heterogeneous, a systematic influence of pharmacological treatment on EEG functional connectivity is most unlikely. A large body of evidence could show that the degree of handedness is a marker for individual differences across various domains including episodic memory retrieval (91). In a volumetric analysis, individuals with less consistent hand preference showed a larger corpus callosum size compared to individuals with a consistent hand preference (92)^3^. According to the hemispheric interaction model (93), a larger corpus callosum size leads to a better interhemispheric interaction. This increased interhemispheric communication might explain the memory advantage in individuals with a less consistent hand preference (91). Since we found networks that contribute to autobiographical memories, future studies should include the degree of handedness as a covariate of no interest in the statistical model to control for the potential influence of handedness on functional connectivity within these networks. As patients improved clinically but substantial trauma-related symptoms persisted, a long-term follow-up study is needed to investigate clinical and neural trajectories across a longer period of time. Future studies should extend the measurement to (different types of) EP. This would further deepen our knowledge how successful treatment effects neural and clinical changes in dissociative parts that react differently to perceived threat compared to ANP. Last, this study measured an overall effect of a multimodal treatment setting. Future studies could include scales that can assess to what extent a particular treatment modality is successful. This could give further insights into which modalities prompt treatment outcome the most.

In conclusion, this work extends a previous emotion regulation functional connectivity study (27) by demonstrating that a phase-oriented treatment is associated with functional connectivity changes in the delta frequency band in networks involved in (emotional) autobiographical memory. Further, patients profited across several clinical domains. These results suggest that treatment provided by therapists who have training in complex trauma and dissociation seems to be beneficial and merits further investigation.

## Data availability statement

The data that support the findings of this study are available from the corresponding author, YS, upon reasonable request.

## Ethics statement

The studies involving human participants were reviewed and approved by Local Ethics Committees of the Canton of Zurich and Thurgau. The patients/participants provided their written informed consent to participate in this study.

## Author contributions

YS conceived and planned the experiment, carried out the experiment, performed the calculations, and wrote the manuscript. CK contributed to data analysis and visualization and interpretation of the data. SB initiated the project. SB, EN, and LJ supervised the project, contributed to the interpretation of the results, and provided critical feedback to the manuscript. All authors contributed to the article and approved the submitted version.

## Funding

This study was supported by Clienia Littenheid AG.

## Conflict of interest

The authors declare that the research was conducted in the absence of any commercial or financial relationships that could be construed as a potential conflict of interest.

## Publisher's note

All claims expressed in this article are solely those of the authors and do not necessarily represent those of their affiliated organizations, or those of the publisher, the editors and the reviewers. Any product that may be evaluated in this article, or claim that may be made by its manufacturer, is not guaranteed or endorsed by the publisher.

## Footnotes

^1^Different subtypes of fragile EPs have to be distinguished (3, 4). To date, only fragile EPs that predominantly demonstrate active, sympathetically driven defense behavior to potential threat have been investigated. Research on fragile EPs that mainly engage in passive, parasympathetically mediated tonic immobility is lacking.

^2^Apart from alterations among dissociative parts, the symptoms are not of a different nature in these three disorders but they are less extreme in DDNOS-1 and even less pronounced in cPTSD compared to DIS.

^3^As an aside, other studies found that corpus callosum size was particularly influenced by factors such as forebrain volume (94) or gender (95). However, a detailed discussion on associations between corpus callosum size and other factors than handedness is beyond the scope of these article.
