# Supplementary material for: Functional connectivity changes in the delta frequency band following trauma treatment in complex trauma and dissociative disorder patients
Source: Front Psychiatry. 2022 Jul 25;13:889560. doi: 10.3389/fpsyt.2022.889560 (PMC9364934; doi:10.3389/fpsyt.2022.889560)
Supplement: Supplementary file 1 [file Data_Sheet_1.PDF]

Supplementary Material

Supplementary Table 1: Overview of inpatient trauma treatment variables and settings per patient (total n = 28)

Participant	Diagnosis	NUMBER	INDIVIDUAL	GROUP	STABILIZATION	NURSING	PHARMA	BODY	MUSIC	ART	OCCUP	COGNITIVE	DBT	ENJOY
1	CDD	1	x	x	x	x	x		x	x				
2	CDD	5	x	x	x	x	x	x	x	x	x			
3	CDD	2	x	x		x		x	x	x			x	
4	CDD	1	x	x	x	x	x	x	x	x	x	x		x
5	CDD	1	x	x	x	x	x	x	x	x				
6	CDD	2	x	x	x	x		x	x	x			x	
7	CDD	1	x	x	x	x	x		x	x	x			
8	CDD	2	x	x	x	x	x	x			x		x	x
9	CDD	1	x	x	x	x		x		x	x		x	
10	CDD	4	x	x		x		x		x				
11	CDD	6	x	x	x	x	x		x	x	x			
12	CDD	5	x	x	x	x		x		x	x			
13	CDD	4	x	x	x	x	x	x	x	x				
14	CDD	2	x	x		x		x		x			x	
15	CDD	1	x	x	x	x		x	x	x				
16	CDD	3	x	x	x	x		x	x	x	x			x
17	CDD	1	x	x	x	x		x			x			
18	CDD	4	x	x	x	x	x	x	x	x				
19	cPTSD	1	x	x	x	x		x		x			x	x
20	cPTSD	3	x	x	x	x		x	x		x			
21	cPTSD	1	x	x	x	x	x	x	x		x	x		
22	cPTSD	1	x	x	x	x		x	x	x	x		x	
23	cPTSD	1	x	x	x	x			x	x				
24	cPTSD	1	x	x	x	x	x	x	x	x				x
25	cPTSD	1	x	x	x	x		x	x		x		x	
26	cPTSD	1	x	x	x	x	x	x	x	x	x			

27	cPTSD	2	x	x	x	x		x		x				
28	cPTSD	1	x	x	x	x		x	x	x				
**Total**	-	-	28	28	25	28	11	24	20	23	14	2	8	5

CDD, complex dissociative disorder; cPTSD, complex posttraumatic stress disorder; NUMBER, number of inpatient stay at the trauma ward (Clienia Littenheid AG); INDIVIDUAL, individual psychotherapy; GROUP, group psychotherapy; STABILIZATION, stabilization groups; NURSING, one-to-one nursing; PHARMA, pharmacotherapy; BODY, body-related therapy; MUSIC, music therapy; ART, art therapy; OCCUP, occupational therapy; COGNITIVE, cognitive training; DBT, dialectic behavioral therapy skills group; ENJOY, enjoyment group. The stabilization groups involve body-related and/or cognitive therapeutic approaches according to the Psychodynamic Imaginative Trauma Therapy (PITT, 1)

Psychotropic medication and comorbid diagnoses

Comorbid diagnoses and psychotropic drugs of the patients were retrieved from their electronic health records (see Supplementary Table 2). Some patients kept benzodiazepine as provisional medication, but none of them took benzodiazepine on a regular basis. In addition, none of the patients under investigation took benzodiazepine at the day of or the day before the EEG experiment.

Supplementary Table 2: Psychotropic medication and comorbid diagnoses of the patients under investigation (total n = 28)

**Psychopharmaca**	**Frequency**
Antidepressiva	19
Neuroleptics	8
Antiepileptics	5
**Comorbid diagnoses**	**Frequency**
PTSD	27
Depression	20
Agoraphobia	1
Panic disorder	1
Obsessive-compulsive disorder	1
Eating disorder	2
Somatization disorder	3
Emotionally unstable personality disorder	2
Mixed or other personality disorder	1

PTSD, Posttraumatic Stress Disorder

Supplementary Table 3: Internal consistency (Cronbach's alpha) of self-report instruments of the participants under investigation (total n = 66)

**Self-report instruments**	**pre**	**post**
PCL-C total	0.97	0.97
FDS	0.97	0.97
SDQ-20	0.92	0.91
PosDiss	0.91	0.90
NegDiss	0.94	0.93
BDI-II	0.96	0.96
STAI-T	0.97	0.94
DERS total	0.98	0.97
ERQ Reappraisal	0.90	0.82
ERQ Suppression	0.82	0.83

pre, pre-treatment; post, post-treatment; PCL-C, Posttraumatic Stress Disorder Checklist, civilian version; FDS, Fragebogen zu Dissoziativen Symptomen; SDQ-20, Somatoform Dissociation Questionnaire; PosDiss, Positive Dissociative Symptoms; NegDiss, Negative Dissociative Symptoms; BDI-II, Beck's Depression Inventory; STAI-T, Stait-Trait Anxiety Inventory; DERS, Difficulty in Emotion Regulation Scale; ERQ, Emotion Regulation Questionnaire. Internal consistency values were calculated as Cronbach's alpha in R using the package psych (2). Missing values were deleted pairwise.

Supplementary Table 4: Specification of ROIs as implemented in the sLORETA toolbox

**BA**	**L/R**	**MNI coordinates (x, y, z)**	**Brain structure**
2a	L	(-55, -25, 50)	Postcentral gyrus (Primary somatosensory cortex)
2b	L	(-45, -30, 45)	Postcentral gyrus (Primary somatosensory cortex)
4a	L	(-35, -25, 55)	Precentral gyrus (Primary motor cortex)
4b	L	(-35, -20, 50)	Precentral gyrus (Primary motor cortex)
5	L	(-15, -45, 60)	Superior parietal lobule (Paracentral lobule)
6	L	(-30, -5, 55)	Middle frontal gyrus (Premotor cortex)
7	L	(-20, -65, 50)	Precuneus
8	L	(-20, 30, 50)	Superior frontal gyrus (dlPFC)
9	L	(-30, 30, 35)	Middle frontal gyrus (dlPFC)
10	L	(-25, 55, 5)	Frontal pole
11	L	(-20, 40, -15)	Orbitofrontal cortex (lOFC)
13	L	(-40, -10, 10)	Insular cortex
17a	L	(-10, -90, 0)	Occipital pole (Primary visual cortex)
17b	L	(-15, -85, 0)	Lingual gyrus
20	L	(-45, -20, -30)	Inferior temporal gyrus (Fusiform gyrus)
21	L	(-60, -20, -15)	Middle temporal gyrus

23	L	(-5, -40, 25)	Cingulate gyrus (PCC)
24a	L	(-5, 0, 35)	Cingulate gyrus (dACC)
24b	L	(-5, 30, 20)	Cingulate gyrus (dACC/rACC)
25	L	(-10, 20, -15)	Subcallosal cortex (sgACC)
27	L	(-20, -35, -5)	Hippocampus
28	L	(-20, -10, -25)	Hippocampus
29	L	(-5, -50, 5)	Cingulate gyrus (PCC)
30a	L	(-25, -75, 10)	Cuneus
30b	L	(-15, -60, 5)	Cingulate gyrus (PCC)
31	L	(-10, -50, 30)	Precuneus
33	L	(-5, 20, 20)	Cingulate gyrus (dACC/rACC)
34	L	(-15, 0, -20)	Parahippocampal gyrus
35	L	(-20, -25, -20)	Parahippocampal gyrus
36	L	(-30, -30, -25)	Parahippocampal gyrus
37	L	(-45, -55, -15)	Inferior temporal gyrus (Fusiform gyrus)
38	L	(-40, 15, -30)	Superior temporal gyrus (Temporal pole)
39	L	(-45, -65, 25)	Inferior parietal lobule (Angular gyrus)
40	L	(-50, -40, 40)	Inferior parietal lobule (Supramarginal gyrus)
41a	L	(-55, -25, 5)	Superior temporal gyrus
41b	L	(-45, -30, 10)	Superior temporal gyrus
42a	L	(-60, -25, 10)	Superior temporal gyrus
42b	L	(-60, -10, 15)	Superior temporal gyrus
44	L	(-50, 10, 15)	Inferior frontal gyrus (Pars opercularis, vlPFC)
45	L	(-50, 20, 15)	Inferior frontal gyrus (Pars triangularis, vlPFC)
46	L	(-45, 35, 20)	Middle frontal gyrus (dlPFC)
47	L	(-30, 25, -15)	Orbitofrontal cortex (lOFC)
2	R	(55, -25, 50)	Postcentral gyrus (Primary somatosensory cortex)
3a	R	(40, -25, 50)	Postcentral gyrus (Primary somatosensory cortex)
3b	R	(35, -25, 50)	Postcentral gyrus (Primary somatosensory cortex)
5	R	(15, -45, 60)	Superior parietal lobule (Paracentral lobule)
6	R	(30, -5, 55)	Middle frontal gyrus (Premotor cortex)
7	R	(15, -65, 50)	Precuneus
8	R	(20, 25, 50)	Superior frontal gyrus (dlPFC)
9	R	(30, 30, 35)	Middle frontal gyrus (dlPFC)
10	R	(25, 55, 5)	Frontal pole
11	R	(20, 45, -20)	Orbitofrontal cortex (lOFC)
13	R	(40, -5, 10)	Insular cortex
17a	R	(10, -90, 0)	Occipital pole (Primary visual cortex)
17b	R	(15, -85, 0)	Lingual gyrus
20	R	(45, -20, -30)	Inferior temporal gyrus (Fusiform gyrus)
21	R	(60, -15, -15)	Middle temporal gyrus

23	R	(5, -45, 25)	Cingulate gyrus (PCC)
24a	R	(5, 0, 35)	Cingulate gyrus (dACC)
24b	R	(5, 30, 20)	Cingulate gyrus (dACC/rACC)
25	R	(5, 15, -15)	Subcallosal cortex (sgACC)
27	R	(20, -35, -5)	Hippocampus
28	R	(20, -10, -25)	Hippocampus
29	R	(5, -50, 5)	Cingulate gyrus (PCC)
30a	R	(25, -75, 10)	Cuneus
30b	R	(10, -60, 5)	Cuneus
31	R	(10, -50, 35)	Precuneus
33	R	(0, 20, 20)	Cingulate gyrus (dACC/rACC)
34	R	(15, 0, -20)	Parahippocampal gyrus
35a	R	(25, -25, -20)	Parahippocampal gyrus
35b	R	(30, -25, -25)	Parahippocampal gyrus
37	R	(45, -55, -15)	Inferior temporal gyrus (Fusiform gyrus)
38	R	(40, 15, -30)	Superior temporal gyrus (Temporal pole)
39	R	(45, -65, 25)	Inferior parietal lobule (Angular gyrus)
40a	R	(50, -30, 45)	Inferior parietal lobule (Supramarginal gyrus)
40b	R	(50, -45, 45)	Inferior parietal lobule (Supramarginal gyrus)
41a	R	(55, -20, 5)	Superior temporal gyrus
41b	R	(45, -30, 10)	Superior temporal gyrus
42a	R	(65, -25, 10)	Superior temporal gyrus
42b	R	(60, -10, 15)	Superior temporal gyrus
44	R	(55, 10, 15)	Inferior frontal gyrus (Pars opercularis, vlPFC)
45	R	(50, 20, 15)	Inferior frontal gyrus (Pars triangularis, vlPFC)
46	R	(45, 35, 20)	Middle frontal gyrus (dlPFC)
47	R	(30, 25, -15)	Orbitofrontal cortex (lOFC)

BA, Brodmann area; L, left hemisphere; R, right hemisphere; MNI, Montreal Neurological Institute; dlPFC, dorsolateral prefrontal cortex; vlPFC, ventrolateral prefrontal cortex; lOFC, lateral orbitofrontal cortex; dACC, dorsal anterior cingulate cortex; rACC, rostral anterior cingulate cortex; sgACC, subgenual anterior cingulate cortex; PCC, posterior cingulate cortex. In the sLORETA toolbox, several BAs have two centroid voxels (specified with a and b). The labels of brain regions are based on visual inspection and the Juelich Histological and the Harvard-Oxford cortical atlases that are integrated in the fMRIB software (http://fsl.fmrib.ox.ac.uk/fsl/fslwiki/Atlases).

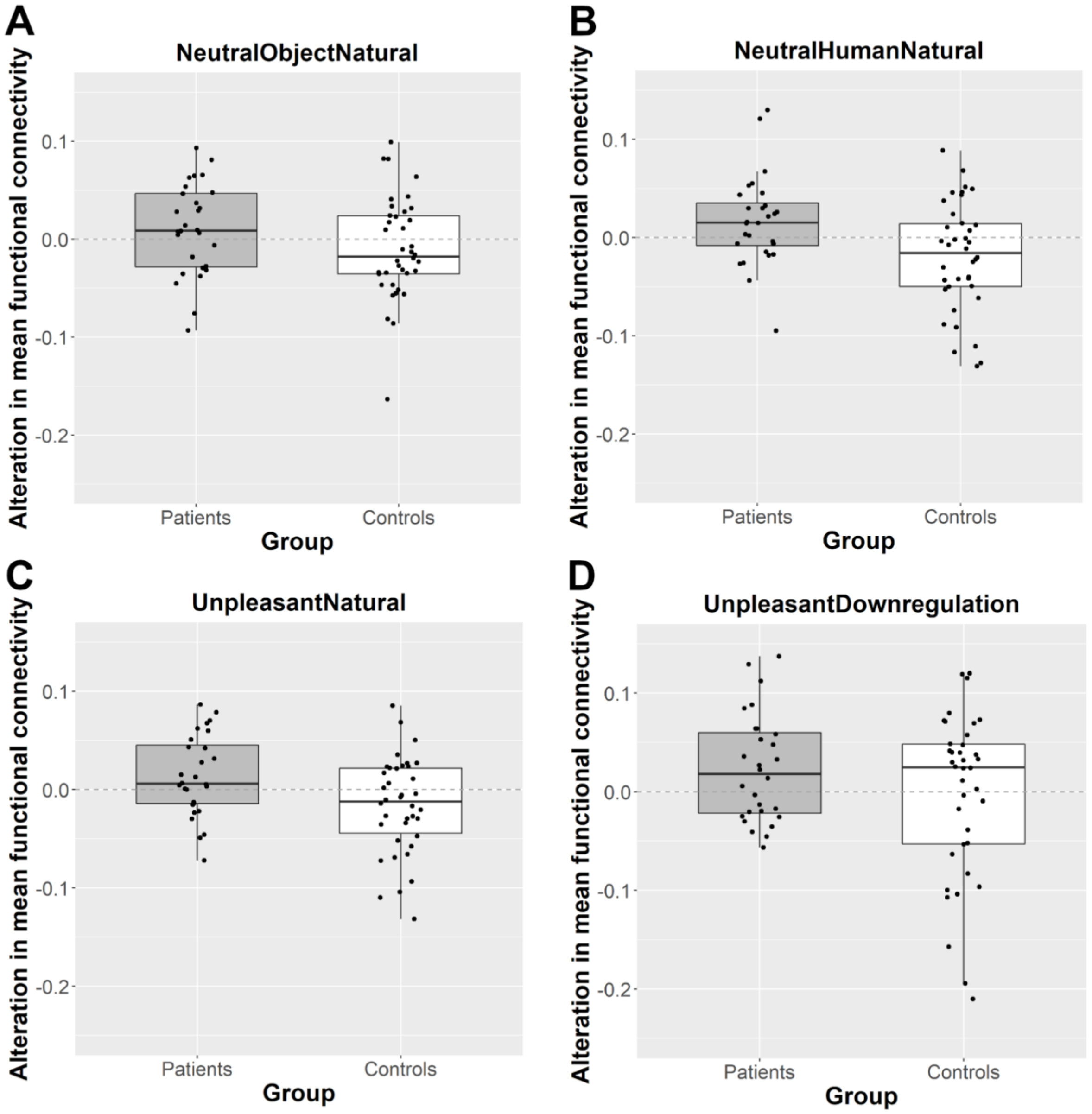

Supplementary Figure 1. Functional connectivity change in the delta frequency band across treatment within the initially impaired network (group x time point interaction) in the A) NeutralObjectNatural, B) NeutralHumanNatural, C) UnpleasantNatural, and D) UnpleasantDownregulation condition. The graphs display the mean functional connectivity per network as revealed by the NBS analysis per group. A positive value indicates higher mean functional connectivity post-treatment compared to pre-treatment, a negative value indicates the opposite. Black dots display individual values. The ends of the whiskers correspond to the lowest and highest values within 1.5 x interquartile range (IQR).

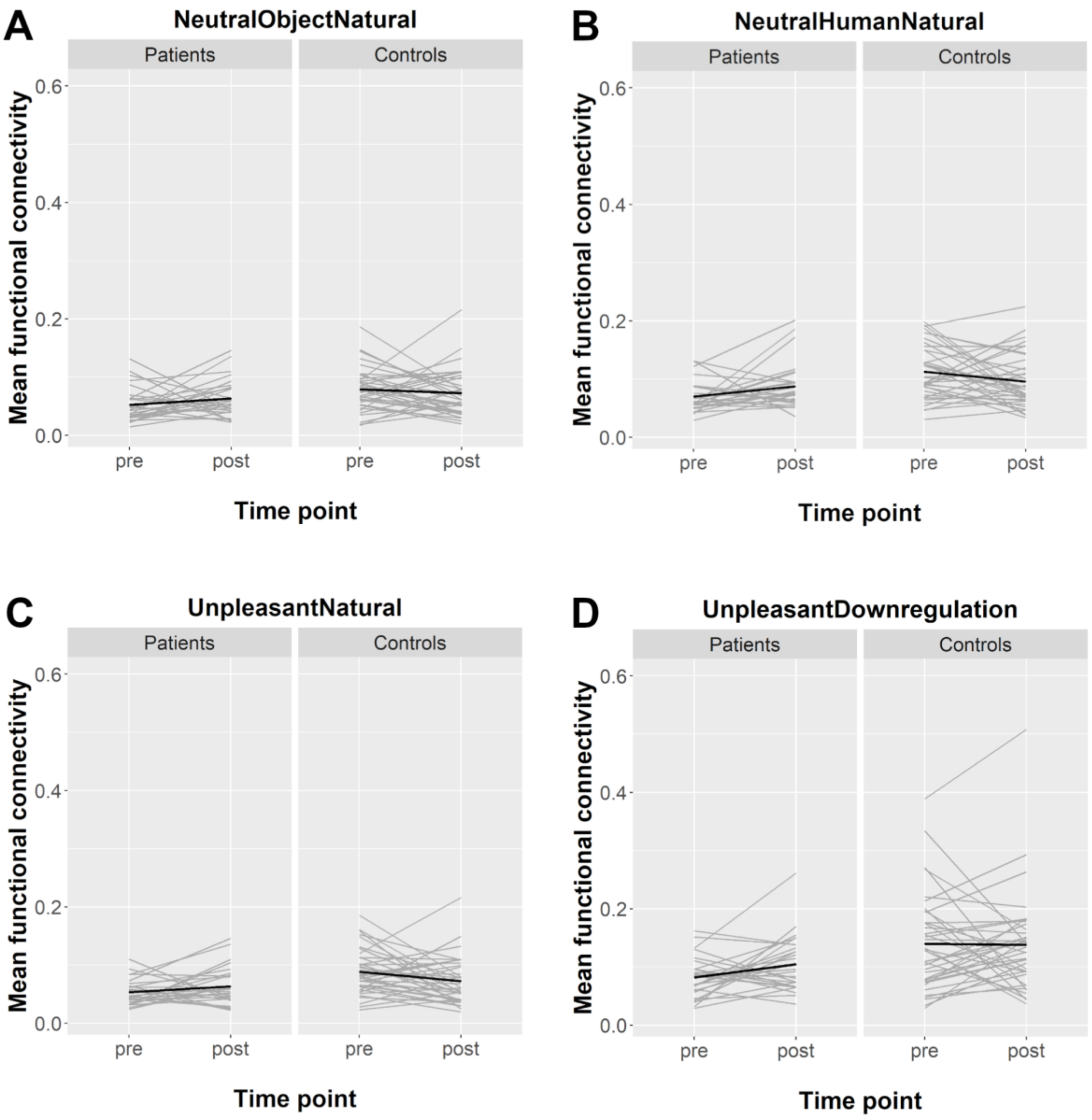

Supplementary Figure 2: Individual trajectories across treatment in the initially impaired delta frequency band networks. The graph outlines the mean functional connectivity per individual at both time points in the A) NeutralObjectNatural, B) NeutralHumanNatural, C) UnpleasantNatural, and D) UnpleasantDownregulation condition.

References

1. Reddemann L. Die psychodynamisch imaginative Traumatherapie (PITT). *Zeitschrift für Psychotraumatologie & Psychologische Medizin* (2003) **1**:1–8.

2. Revelle W. *psych: Procedures for Psychological, Psychometric, and Personality Research*. Northwestern University, Evanston, Illinois (2022).
